# Transition of Adolescents with Inflammatory Bowel Disease—Are the Pediatric Teams up to the Task?

**DOI:** 10.3390/children8100900

**Published:** 2021-10-09

**Authors:** Mette Hald, Katrine Carlsen, Vibeke Wewer

**Affiliations:** Department of Pediatrics, Hvidovre University Hospital, Kettegaard Alle 30, 2650 Hvidovre, Denmark; mette.hald.01@regionh.dk (M.H.); Anne.Vibeke.Wewer@regionh.dk (V.W.)

**Keywords:** transition, transfer, inflammatory bowel disease, children, adolescents, parents

## Abstract

Our aim was to describe the challenges of the transition process of adolescents with inflammatory bowel disease (IBD), as seen from a pediatric-care perspective, to clarify the key obstacles and propose how to move forward. Semi-structured individual interviews of pediatric gastroenterologists and nurses were analyzed and interpreted according to the principles of social science. It is a challenge for the pediatric team to continuously match, support, and gently guide the ever-changing adolescent patient. All adolescent patients should be offered guidance regardless of their starting point, and specific individual needs should be taken into consideration. Adolescents burdened by psychosocial challenges require extra support. Early and continued interdisciplinary effort is essential. Collaboration with the parents must be continuously adapted to prepare them for their new roles and responsibilities. The shift from the pediatric family-focused approach to the individualistic approach of the Adult Gastroenterology Department signifies a fundamental change. Equipping adolescents with disease-management skills remains a comprehensive task. The following crucial questions remain: Who is capable of performing the transition? Who can dedicate the necessary resources for performing the transition? A transition center led by both pediatricians and adult gastroenterologists in an interdisciplinary setting that includes transition-trained persons may be the solution.

## 1. Introduction

The prevalence of inflammatory bowel disease (IBD), which includes Crohn’s disease and ulcerative colitis, is increasing worldwide. Approximately 20 percent of patients develop the disease before they are 18 years old [1,2], and they are therefore initially under the care of the Department of Pediatrics. It is expected that the pediatric team prepares adolescent patients for their transfer to the Department of Adult Gastroenterology at the age of 18. The important skills that adolescents are expected to acquire before the transfer are extensive and involve: (a) disease knowledge; (b) disease self-management; (c) the management of social challenges; and d) the ability to take part in health-related decisions in cooperation with the provider [3,4]. However, four factors—rapid and continuous change during adolescence; individual starting points; psychosocial factors; and parental roles—greatly influence how quickly and successfully adolescents can obtain the skills needed to meet the demands of the Adult Gastroenterological Department, and why the level of preparedness at transfer may vary considerably [5]. The number of skills to be learned as well as the differences between the two departments must also be taken into consideration. Obstacles related to the transition process are multifaceted and despite increasing awareness of the need of transitional care [6], no “golden standard” for transition currently exists. The primary aim of our study was to describe the challenges of the transition process of adolescent IBD patients, as seen from a pediatric perspective, to clarify the key obstacles and propose how to move forward.

## 2. Materials and Methods

Statements from semi-structured interviews with pediatric gastroenterologists and nurses were analyzed and interpreted according to the principles of social science. The 30 min interviews were conducted individually. The semi-structured interview was designed by the first author. The questions were constructed from daily clinical experience and relevant literature [7,8,9,10]. The method of the semi-structured interview was chosen because it is the most frequent source of qualitative data in health services research. The method is dialogue-based and led by an interview-guide supplemented by follow-up questions, probes, and comments [11]. The interview guide is presented in Table 1. Interviews were taped, transcribed, translated from Danish to English, and sent to the interviewees for approval. The analysis of the statements included a description of the topics and the perspectives of the interviewees. The main points of the article are illustrated through excerpts from the interviews.

The interviewees consisted of three pediatric gastroenterologists and two IBD nurses from the Department of Pediatrics, Hvidovre University Hospital, Denmark. The interviewer was a health psychologist from same department.

No approval was obtained from the National Ethics Committee or the Data Protection Authority as the manuscript does not contain patient-related data or sensitive personal data.

## 3. Results

The results from the interviews are thematically presented with examples from the answers provided during the interview, followed by an analysis of the related challenges and recommendations.

**#1 A fundamental premise and a challenge: A pediatric patient is ever-changing and maturing—an active and individually adapted approach is thus required.**

Pediatric gastro-nurse 1 (PGN1): *“Adult patients are quite static, whereas children are undergoing a continuous developmental process.”*

Pediatric gastroenterologist (PG) 1: *“We need to do more than just support (their independence)—we also have to push them towards it (…), give them a chance to manifest their independence in relation to their parents at an earlier stage than they have done so far (…).”*

Challenges: Adolescent patients are complex—not just because the disease is often more widespread and aggressive in children/adolescents than in adults, but because these adolescent patients are constantly developing and changing physically, cognitively, and emotionally. They may take developmental quantum leaps between scheduled consultations. Developing their gradual independence is pivotal and requires special attention and support.

Recommendation: It is a key challenge for any pediatric team to continuously adapt their approach and pace to match, support, and gently challenge the ever-changing adolescent patient. Improving the transition and transfer requires the prioritization and the availability of education and resources.

**#2 Recognizing and acknowledging the differences: The distance to and speed at which one can reach the finish line is defined by the individual’s circumstances.**

PG1: *“They have been—and some still are—in the process of taking charge of their disease, some of them successfully, others less so. The variation is considerable, some of them are very mature, fast learners, and capable, while others are very hesitant learners, and some are held back by overly caring parents.”*

Challenges: The evolving independence of the adolescent patient is influenced by individual factors such as their personality, resilience, and the severity of the illness, as well as the quality of the interaction between the adolescent and their parents, and—to a certain extent—the demands put forward by the hospital. Differing levels of independence and maturity mean that individual needs during the transition process vary considerably from patient to patient. As all patients have different starting point, it is difficult to imagine that all will reach the set goals at the same time.

Recommendation: “Don’t focus on the type of disease afflicting the patient—instead focus on what type of person is afflicted by the illness” (quote attributed to A.R. Luria). All adolescent patients should be offered guidance and support regardless of their starting point. Some will need more time and support than others when it comes to mastering independent disease management.

**#3 Acknowledging vulnerability: Adolescents burdened by psychosocial challenges require extra support.**

PG1: *“We see patients who—initially or subsequently—are seriously affected by mental problems or psychiatric illness that leads to a very problematic transition process. And we see the patients from low-functioning families, where the process to a worrisome degree is dependent on the maturity of the individual patient.”*

PG2: *“One of our biggest challenges is dealing with the patients who have substance abuse or who develop mental disorders. That is very demanding, and we may not even discover them all. It is my impression that there is an overrepresentation of adolescents with these types of problems.”*

Challenges: Socio-demographic factors, family dynamics, and individual variables are of fundamental importance to the risk and protective factors influencing a patient’s life. These factors are in play at the individual level (i.e., intelligence, mental resilience, illness, social skills) as well as in the immediate environment (i.e., family communication, organization, emotional warmth), and greatly influence the adolescent’s resilience and ability to cope with the disease and with life in general [12].

Children and adolescents (17.3%) suffering from IBD are more at risk of depression, anxiety, and social isolation than their healthy peers, and a special concern is an increased risk of suicide [13,14]. In addition, some have pre-existing challenges such as ADHD and autism [14]. These factors may all be detrimental to the adolescent’s ability to cooperate and comply with the required treatment. This in turn increases the risk of “getting lost” in the transfer from pediatric to adult health services which may compromise their future treatment and health.

Recommendation: These vulnerable adolescents need extra support, attention, and follow-up, and they do not necessarily fit into the established routines preceding or following the transfer. Early and continued interdisciplinary efforts carried out in close cooperation among doctors, nurses, social workers, psychologists, and the social services in municipalities are essential and necessary.

**#4 The necessary changes in partnership: Collaboration with the parents must be continually adapted.**

PGN1: *“I am not sure that the parents even see it. They see that it is important to be there for their child, but they don’t see that it is even more important that they step back, because they are so enmeshed, and they find it very difficult to separate themselves from their children.”*

PG2: *“The parents are very committed, especially if the adolescents have had many symptoms through the course of their disease (…) The adolescents who have had an easy run disease-wise will gradually become independent—they will come to manage the consultations on their own and so on. The ones who have had a difficult course…well, a kind of symbiosis seem to grow in the instances where the child has been seriously ill.”*

Challenges: Pediatrics is characterized by close collaboration among staff, parents, and the patient. As the patient approaches adolescence, the pediatrician and staff must shift their focus in order to develop an increasingly independent and engaging relationship with the young patient. Consequently, the parents will have to gradually step back. This is challenging for some. At best, a parallel process will emerge, allowing the adolescent to gradually take over while the parents slowly let go. However, parental overprotection of chronically ill children/adolescents is a well-known phenomenon.

Furthermore, an uneven course of development is often seen among adolescent patients, and the adolescent drive towards independence may be delayed. Early disease onset and the complicated impact of medical care on family dynamics placing parents in a pivotal role may make it even more difficult for them to adjust and relinquish responsibility [15].

Recommendation: The shift from the pediatric family-focused approach to the individualistic approach of the Adult Gastroenterology Department signifies a fundamental change in the parental role. Thus, not only the adolescent but also the parents need to be guided and equipped for their new roles and responsibilities [16]. Without preparation, the parents may not be the supportive force in the transition and transfer process that they otherwise could be.

**#5 Helping the adolescent acquire the necessary skills: Training and teaching disease-management skills to adolescents is a comprehensive and demanding task.**

A well-planned transition program may help the adolescent patient acquire the skill set necessary for successfully managing the disease, e.g., knowledge and understanding of the disease and treatment; confidence in their own ability to handle challenges (self-efficacy); the ability to make decisions and solve problems; speaking up for oneself (self-advocacy); and information gathering [17]. Some of these skills emerge in line with the ever-evolving maturity of the adolescent, whilst others have to be actively encouraged through the efforts of the pediatric team. The four following challenges (1–4) were identified:

Challenge 1—Planning:

PG1: *“It has to be planned in advance, and we need a blueprint (…). It needs to be organized and systemized to avoid it being pushed to the lowest priority when other pressing matters have to be attended to.”*

PG3: *“I believe it is important to standardize, in order for you to know what is expected. We have all contemplated how this could be done, but I’m not sure that we have reached the same conclusion.”*

Integrating various supportive and educative initiatives into the busy and hectic daily life of a Department of Pediatrics is challenging and requires a clear structure and shared understanding of the methods and goals. This is an ongoing challenge that needs to be seriously addressed.

Challenge 2—A multifaceted focus:

PG1: *“They feel stabbed in the back by the disease when it comes to their hopes of living a teenage life resembling that of their peers. They all have a built-in frustration and disappointment (…). They have a chronic disease, and on top of that they usually also carry a load of derived consequences, namely the social and psychological challenges. This holds true for most.”*

PG1: *“A more holistic approach will be under constant pressure, because (…) a pediatric consultation is scheduled to last a certain number of minutes, and a broadened focus is time-consuming. It will take a lot of work and planning if it is to be done properly.”*

Anxiety, depression, and lack of confidence and knowledge constitute obstacles to a successful transition and transfer. These factors have to be taken into account [15]. An expanded consultation focus is needed which includes not only somatic but also psychosocial wellbeing in the overall assessment of the patient.

To support the development of the adolescent’s independence, in addition to consultation with a medical doctor, it may also be beneficial to consult a transition-trained person, with whom priorities, treatment goals, and youth-related topics and issues [2] can be discussed. Furthermore, it would be beneficial for the adapted and innovative approach to include and promote an interdisciplinary support (i.e., psychologist, dietician, social worker, relaxation, and psychomotor therapist).

Challenge 3—Moving from a triadic to a dyadic collaboration:

PGN1: *“For some, it (one-to-one consultation) comes naturally, and for quite a lot it does not—at all. In those instances, we have to take charge indicating “this is how it is done” and guide them (the parents) a bit.”*

PG1: *“We are far from reaching the goal of every adolescent being trained in individual consultations without parental participation prior to transfer.”*

It is important to gradually enable and encourage the adolescent to independently manage their own consultations with the doctor or nurse.

Challenge 4—Continuity:

PG3: *“It is actually quite difficult to arrange to see the same patients throughout. In my opinion, the 15, 16, and 17 years old would benefit from having a set group of health professionals.”*

PG3: *“Continuity is important, especially for the chronically ill. It’s not about preferring one specific doctor. It is about seeing the same doctor, and maybe spending the first four consultations getting ready to tell—and then finally you do (…). It takes time for her/him to open up, and if you (the patient) are introduced to a new person every time, you will never reach that point.”*

A cornerstone in building trust and fostering cooperation is having continued contact with a select group of doctors and nurses throughout the transition process.

Recommendations (1–4): To truly recognize the extent and the importance of the task and acknowledge the amount of resources and skills that are needed to do the job.

**#6 The lack of core communalities and communication: The two departments are different and have limited knowledge of each other’s practices and challenges.**

PG3: *“I have worked both places, and I believe that a key challenge is the very sudden change caused by the fact that things are done so very differently (by the Adult Gastroenterology Department). The consultations are shorter, you are expected to be prepared, and to manage your medication and prescriptions. There is no safety net in the sense that if you neglect to do stuff, it might not be picked up on, as we would do (in the Pediatric Department). I would say that the differences are huge (…). The challenge is to create some sort of bridge so that the patients gradually are led to see “this is where we are going”, and that those who are welcoming their new patients somehow accept and adapt to the fact that “all right, this is the material we have received, and we are going to shape them. As they are “now in our care, we’ll have to spend some time shaping them to fit into our way of doing things”.*

PG2: *“I don’t know how they are doing, once they are transferred”*

Challenges: In adult care, the emphasis is on the somatic aspects, and the patient is expected to be self-reliant and capable of efficient disease management. The transfer from pediatric to adult care therefore signifies not just a change of location, but a change in culture, values, and expectations. Due to the significant differences between the two departments, adolescents will be limited in their opportunities to draw on their previous experiences and the strategies developed during their time in pediatric care [18]. Part of the transition preparation must therefore focus on the impending changes. The differing approaches of the two departments are to a large extent defined by the fact that the care of a pediatric patient differs from that of an adult patient, and vice versa. This is reflected in the culture of care as well as by the provider’s perspective, competency, and attitude [19]. The departments do not necessarily have much knowledge of the approach, values, and ideas held by the other. Their dissimilarities in terms of approach and thinking may cause division, making it difficult for them to converge for the good of the patient [20], and may result in diverging opinions on how to best prepare the adolescent for transfer [21].

The lack of systematic follow-up and feedback means that the pediatric team does not have an overview of the efficacy of the preparatory transition efforts and the specific challenges of the transfer. This makes it difficult to determine whether the assessments of transition readiness hold true, and challenges to the current approach may be overlooked.

**Figure 1:**
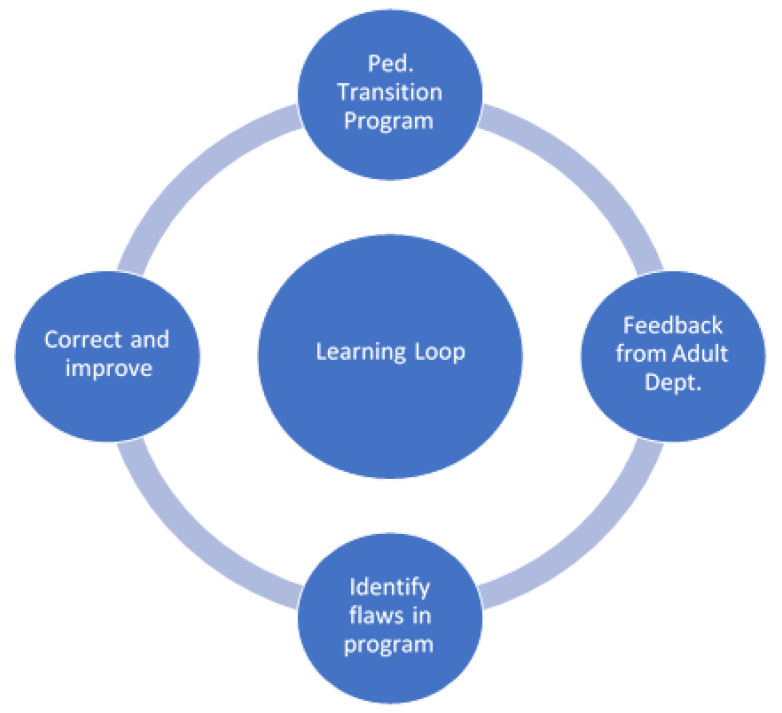
Learning loop inspired by Hansen (2018) [22]. Adapted with permission from the author Hansen, M.T whom holding the copyright, published by Simon & Schuster 2018).

Recommendation: Transfer experiences may be used to develop the practice through a “learning loop” [22], illustrated in Figure 1, where systematized feedback from the Adult Gastroenterology Department and previously transferred adolescents uncover the shortcomings and failings of the approach, thereby making it possible to make improvements [23].

## 4. Discussion

It is a key challenge for the pediatric team to adapt the approach and pace to match, support, and gently challenge the ever-changing adolescent patient. All adolescent patients should be offered guidance and support regardless of their starting point, and the focus must be on the specific needs of the individual patient. Furthermore, adolescents burdened by psychosocial challenges require extra and different support. As pointed out by Philpott [2] and others, early and continued interdisciplinary effort carried out in close cooperation among doctors, nurses, social workers, psychologists, and the social services in municipalities is essential. Collaboration with the parents must continually be adapted, and thus, not only the adolescent but also the parents need to be guided and equipped for their new roles and responsibilities.

Prioritization, education, and resources are needed to facilitate the transition and transfer. The pediatric team is continuously seeking to strengthen and develop their transition efforts in terms of their structure, content, and interdisciplinarity. However, time restraints, lack of continuity, the need for the specialized training of healthcare workers, and the absence of an overall plan all complicate their implementation into the daily work routine.

**Figure 2:**
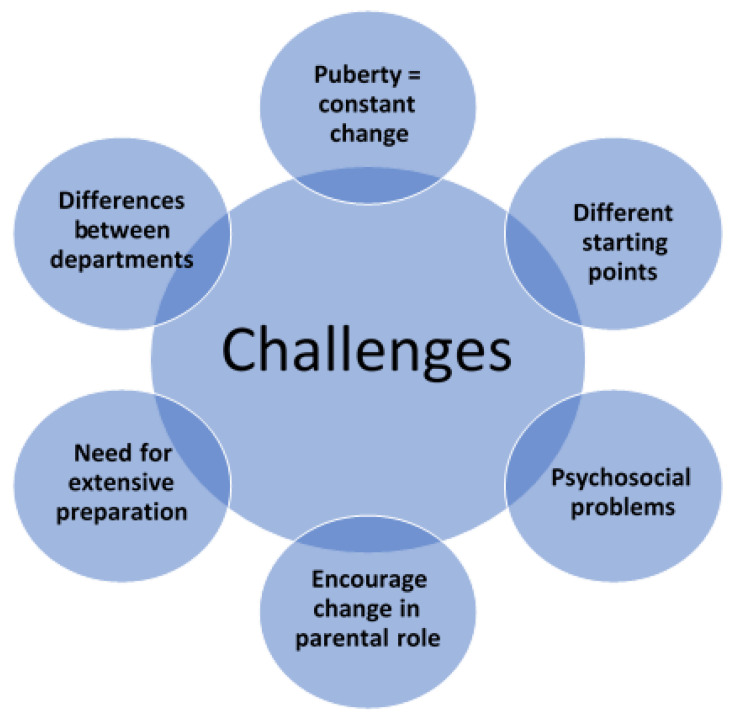
Challenges of the transition process.

Despite the aforementioned challenges acknowledged in the literature as well as by international organizations (e.g., European Crohn’s and Colitis Organisation) and the different transition approaches which have been suggested, no consensus on a transition model exists [3,6,24,25]. This might be due to the challenges being multifaceted (Figure 2). The diversity of and differences among adolescent patients mean that the transition model needs to be more refined than a “one-size-fits-all” approach. The same goes for “one expectation fits all”—this also needs to be based on the specific circumstances of individual patients. Variation in starting points and resources means that some are ready for an early transfer, while others need an extended preparation strategy. This suggests that the individual’s level of maturity and competencies may be more relevant for the transfer planning than the actual age of the patient.

Studies have shown that another key factor in creating a successful transfer is the heightened awareness of the demands and expectations that await the adolescent, specifically defining the skills and behaviors needed to perform and thrive in the new surroundings [17,18]. Close collaboration and exchange of knowledge between the two departments is therefore of the utmost importance and will hopefully lead to a dynamic and flexible transition process that actively supports and develops the competencies of the adolescent.

The strengths and limitations of our analysis are as follows. The semi-structured approach allowed the interviewees to expand on topics and include whatever they found to be of significance. As pointed out by De Jonckheere and Vaughn, semi-structured interviews are an effective method for data collection when the researcher wants to collect qualitative open-ended data and explore participants’ thoughts and beliefs about a particular topic [11]. However, the authors selected the topics for the interview guide and no validation or Delphi process was performed. It is therefore not certain that the questions were applicable to the topic and there may be a risk that other important issues were overlooked. The transition process involves four stakeholders—the patient; the parents; the pediatric gastroenterological team; and the adult gastroenterologists—and our interviews only provided information from the context of the experiences of the pediatric gastroenterological team. We have previously presented the challenges and obstacles in the transition process through the eyes of the gastroenterologist [5]; however, interviews with the patients and parents would contribute to a deeper understanding of the interconnectedness and different needs arising from the transition. It is also a limitation that this study was confined to a single hospital setting. Expanding this research to more hospitals may broaden our knowledge and deepen our understanding of both the common denominators and the variations in the field of transition and transfer.

In conclusion, we found that the challenges experienced by professionals are related to structural issues (e.g., time restrictions, planning difficulties, lack of collaboration across departments) as well as the characteristics and diversity of the patient group (different starting points and capabilities, the degree of parental involvement, and vulnerabilities caused by psychosocial challenges). These findings are consistent with other studies within the field [6].

The following critical questions remain: who is able to, who possesses the competencies to and who can dedicate the resources to educating and guiding the patients and the families? This question was also raised by Sattoe et al., who in their paper evaluated the value of a transition clinic and concluded that organizational and financial barriers need to be addressed before guidelines and consensus statements in healthcare policy and daily practice can be effectively implemented [24].

The differences between the pediatric and adult departments with regard to their approaches and values are well described [5,18,19,20] and may be so deep-rooted that a stepping stone between the departments is urgently needed to optimize and safeguard the transition and transfer of the adolescent patient. The need to bridge the gap and create a stepping stone for adolescent patients with IBD suggests that the creation of an interdisciplinary setting where pediatricians and gastroenterologists join forces in a transition center may be the best way forward. Such a center would build on the strengths of both departments and equal attention would be given to support as to autonomy. A stepping stone center built on an approach of “support and autonomy” has the potential to bridge the gap and strengthen the independence and confidence of adolescents suffering from IBD within a safe environment.

## Figures and Tables

**Table 1:** Interview guide.

Topics	Questions
**The patients and parents**	- Do you find that there are common features that characterize this group of patients? - What is your impression of the role and approach of the parents?
**Challenges**	- What do you see as the hardest challenges for the patients in connection with transfer? - What are the most difficult challenges for the department in relation to this group of patients? - What characterizes a successful/unsuccessful transition and transfer? - Which elements do you consider crucial for a successful transition and a good process going forward?
**Interventions**	- What should be given priority in the department’s transition work? - What knowledge/skills will the adolescent patient need to master before transfer? - Do you find that there are shortcomings in the current approach? Which ones? - How do we best support the group of patients who have disease onset + 16 years of age? - Do you have other suggestions/ideas?

## Data Availability

Data supporting reported results can be found by contact to the authors.
